# Operation for Typhoid Ulcer

**Published:** 1901-01-12

**Authors:** 


### OPERATION FOR TYPHOID ULCER.

Dr. Elliot,[1] surgeon to the Massachusetts general hospital, reports a case in which he operated successfully on an ulcer of the intestine occurring in a case of typhoid fever. The patient in the third week of typhoid fever had been attacked with a sudden severe abdominal pain four hours previously. He was still suffering greatly, in spite of a small opiate. The abdomen was moderately distended, rigid, and tympanitic. There was no dulness, and nothing could be detected by palpation except a general tenderness. The patient was evidently very ill. A laparotomy was done at once. The peritoneal cavity was found filled with a turbid fluid containing white fibrinous flakes. The gut was darkly reddened and congested, and several patches of adherent greenish white exudation were seen on the intestines, which, however, were not glued together. On tracing up the bowel, about four feet from the cæcum the ileum was found to be covered with a grey fibrinous mass. On withdrawing it and packing it around with gauze, an ulceration was plainly to be seen on the side opposite the mesentery. The centre of this ulcer was just ready to slough out. It was difficult to see how much leakage could have taken place, and gentle pressure failed to force the fæces through. Other small patches of fibrin were seen on the bowel, but no other ulceration was discovered. Two layers of Lembert's sutures were applied, and the abdomen was carefully wiped out. A small drain of gauze was left, leading down to the line of sutures. On the third day the bowels were moved by an enema, and on the fourth the drain was removed. The patient recovered very well, although he had a relapse of the fever a week after the operation. This case illustrated a point which has before been pointed out in these columns, namely, that the onset of pain and symptoms of peritoneal infection does not of necessity mean that a typhoid ulcer has so completely given way as to lead to extravasation of the bowel contents. There is a period in many cases at which the ulcer gets so near the surface as to set up a septic peritonitis without causing actual extravasation, and this condition may last for several hours before the final catastrophe occurs. Needless to say that this is the very moment when an operation may be done with the greatest hope of success.

[1] New York Med. Record, Dec. 22.

